# Robert Hertz, Anthropophagic Practices and Traditional South Fore Mortuary Rites in Papua New Guinea

**DOI:** 10.1177/00302228241239210

**Published:** 2024-03-08

**Authors:** Jerome T. Whitfield, Wandagi H. Pako, Michael P. Alpers

**Affiliations:** 1 UCL Institute of Prion Diseases, 47973 MRC Prion Unit at UCL, London, UK; 2 116896 Papua New Guinea Institute of Medical Research, Goroka, Papua New Guinea; 3 168274 Curtin University, Perth, Australia

**Keywords:** mortuary rites, south fore, Robert Hertz, Daniel Lagache, cannibalism, transumption

## Abstract

In this paper we examine the traditional mortuary rites of the South Fore people of Papua New Guinea using Robert Hertz’s theory of secondary burial and the three mechanisms of mourning identified by Daniel Lagache. The ethnographic data that we obtained on South Fore interpretations of their own mortuary rites showed that all forms of corpse handling achieved the same end results through the process of secondary burial. Furthermore, the three mechanisms of mourning applied equally to all forms of corpse disposal and we found no evidence to support psychosexual interpretations of mortuary anthropophagy which emphasise aggression. South Fore interpretations of mortuary anthropophagy show that the dead were eaten out of love, and to protect the mourners from the painful emotion of witnessing the decomposition of the corpse. These findings affirm the relevance of the concepts of Hertz and Lagache to the universal human experiences of death and mourning.

In this paper we examine ethnographic data on the traditional mortuary rites of the South Fore people of the eastern highlands of Papua New Guinea and show that all forms of disposal of the dead, including mortuary anthropophagy, burial, platforms and baskets accomplished the same final results through the process of secondary burial, as described by Hertz (1960). The relationships of identity and ambivalence between the mourners and the deceased, and the unacceptability of death, were the same for all forms of corpse disposal, and, like Hertz (1960), Lagache (1993) and Conklin (2004), we reject psychosexual interpretations of mortuary anthropophagy which emphasise aggression (Gillison, 1993; Sagan, 1974; Sanday, 1986; Stephen, 1998) and understand mortuary anthropophagy, through indigenous interpretations, to be the favoured means of disposal of the body of a loved one among the South Fore.

Hertz’s essay entitled ‘Death and the Right Hand’ (1960), first published in 1907, remains the most significant historical and theoretical contribution to the sociological study of death (Venbrux, 2007, p. 6). Based on his analysis of ethnographic data, mainly about the Dayak of the Malay Archipelago, he was able to divide mortuary rites into two phases: the intermediary period and the final ceremony. During the intermediary period, which started at the moment of death and lasted till the final ceremony, the corpse was placed in a temporary sepulture where the bones and flesh separated, and the mourners and souls of the deceased underwent a gradual transition in their condition. In the final ceremony the bones, now purified of the putrefied flesh, were placed in their final sepulture, the souls of the deceased were reunited in the land of the ancestors and the mourners were reintegrated into society (Hertz, 1960, p. 54). Hertz also extended his theory of secondary burial to other forms of corpse disposal including mortuary anthropophagy. He described endocannibalism as ‘a sacred meal’ in which specific groups of a society took part.

By this rite the living incorporate into their own being the vitality and the special qualities residing in the flesh of the deceased; if this flesh were allowed to dissolve, the community would lose strength to which it is entitled…. at the same time, endocannibalism spares the deceased the horror of a slow vile decomposition…. (Hertz, 1960, p. 44)

Although Hertz’s work was a sociological discourse a psychological theme ran through it, and this theme was expanded by the French psychiatrist Daniel Lagache (1993) in his paper entitled ‘The work of mourning: ethnology and psychoanalysis’, first published in 1938. Freud, in ‘Totem and Taboo’ (first published in 1912-13), identified the ambivalence of mourners’ emotions, and not long afterwards he expanded on the themes of mourning and its psychopathological forms in his work ‘Mourning and melancholia’ (first published in 1915) (Freud, 2005). Ambivalence was the unconscious conflict between the loving and hostile emotions of the mourner which manifested as the conscious grief and the unconscious satisfaction at death. Lagache identified a strong similarity in ‘the work of mourning’ between traditional societies, based on the work of Hertz, and individuals in Western society, based on the work of Freud (Lagache, 1993). In Hertz’s work he noted the unacceptability of death, ambivalence in the unconscious aggression of the mourners towards the deceased, inverted to the aggression of the dead towards the living, and how identity was shared between the closest relatives and the deceased. Lagache considered endocannibalism in terms of identity and aggression, identity through absorbing the deceased and their qualities and placing them in an honourable sepulture, and ambivalent aggression through destruction of the corpse (Lagache, 1993, p. 27). However, he was cautious about the link between endocannibalism and aggression, noting Hertz’s rejection of the idea that it might be related to a “refinement of cruelty or the satisfaction of a physical appetite” (Hertz, 1960, p. 44).

Hertz’s rejection and Lagache’s caution about the link between aggression and mortuary anthropophagy was shared by Beth Conklin, whose ethnography on Wari mortuary anthropophagy in South America emphasises indigenous interpretations (Conklin, 2004, p. 245). Wari mortuary anthropophagy was based on reciprocal predation between humans and their ancestors and allied spirits with whom they intermarried (Conklin, 2004, p. 251). The ancestors were re-embodied in peccaries and sought out kin hunters so they could provide food for their loved ones. Anthropophagy marked the metamorphosis of the deceased from the living to the dead, from meat eater to meat provider, from predator to prey (Conklin, 2007, p. 1257). The Wari explained to her how anthropophagy helped the mourners overcome their grief for the loss of a loved one by altering their memories of, and emotions towards, the deceased. The Wari also emphasised their preference for being incorporated into the bodies of relatives to being left to rot in the earth, a preference shared by other South American societies which practised mortuary anthropophagy in the past (Conklin, 2004, p. 247). She noted a clear connection between Wari anthropophagy and the work of mourning, described by Freud as the gradual detachment from the memory of the deceased (Conklin, 2007, p. 1259). Furthermore, the Wari rejected the idea of “any form of overt, covert, or displaced hostility” during mortuary anthropophagy (Conklin, 2004, p. 245).

Melanesian reports and theoretical interpretations of mortuary anthropophagy have focused on the eastern highlands of Papua New Guinea (Gillison, 1993; Lindenbaum, 2013; Meigs, 1984; Sanday, 1986; Stephen, 1998; Strathern, 1982). Bloch and Parry suggested that mortuary anthropophagy benefited those who consumed the body by transferring biosocial substances, substances which were limited in quantity, from the deceased to the living (Bloch & Parry, 1982, p. 8), evidence of which was implicit in Strathern’s examples of Melanesian anthropophagy among which only the Gimi practised mortuary anthropophagy (Strathern, 1982). In many cases the resources perceived by a society to be most vital to the social order were revitalised during mortuary rites (Bloch & Parry, 1982, p. 7), and, in the case of the Gimi, Strathern concluded that women’s anthropophagic practices reproduced the clan (Bloch & Parry, 1982, p. 8). However, Bloch and Parry cautioned that the regulation of bodily substances during life and the concept of life as a limited good transmitted during mortuary rituals were not necessarily analogous, and needed to be investigated on an individual basis (Bloch & Parry, 1982, p. 9).

Strathern (1982) linked the lack of pigs in highland fringe areas and marriage patterns that turn back as concomitants for anthropophagy in the eastern highlands. However, the ecological and social concomitants were opinions: fringe groups, including the Gimi and Fore, had pig herds which enabled individuals and lineages to fulfil their social obligations (Gillison, 1993, p. 253n3; Lindenbaum, 2013, p. 39), and although the ideal marriage was to existing affines, the constant warfare and distrust experienced by these societies resulted in groups seeking marriages from multiple affines (Gillison, 1993, p. 31; Lindenbaum, 2013, p. 40).

Meigs (1984) explored Hua rules surrounding the consumption of food and the social relationships between givers and recipients. According to Meigs, Hua religion was based around the regulation of an individual’s vitality called *nu*, which included all bodily substances. The negative form of *nu* was known as *siro na* and the positive as *auna*; *siro na*, defined as pollution (Meigs, 1984, p. 99), was removed from the body to stop its debilitating effect whenever possible; *auna*, defined as the ‘positive or nourishing aspect of *nu*: shadow or spirit’ (Meigs, 1984, p. 179), was collected and protected whenever possible (Meigs, 1984, p. 135). The transfer of positive or negative *nu* depended on the relationship of the giver to the receiver and their sex.

Meigs’ fieldwork was conducted 20 years after the cessation of mortuary anthropophagy and she noted considerable embarrassment amongst the Hua when she asked questions about the practice; consequently she did not intentionally collect data on the topic (Meigs, 1984, p. 164). Furthermore, her work contains no mention of mortuary rituals. It was on the basis of food rules that Meigs understood Hua anthropophagy (Meigs, 1984, p. 40).

In Sanday’s (1986) interpretation of Meigs’ work she understood *nu,* which originated from the human body, as part of a theory of life and death, and male and female: consequently she viewed anthropophagy as essential for both social and biological reproduction of Hua society. She interpreted Hua anthropophagy as “hunger for vital essence and social continuity” (Sanday, 1986, p. 67).

Sanday, drawing on both Jung and Freud, suggested a connection between the image of vital essence (*nu*) and the flow of intrauterine feeding, and suggested that the fears attached to female fertile substances projected an image of a weak ego and a dominant female figure (Sanday, 1986, p. 47). For the Hua, the primal mother of their origin myth, the maternal uroboros, was a self-sustaining entity, symbolising the merging of psychological, physical and alimentary processes in which all opposites originated. She understood this as corresponding with Freud’s oral cannibalistic phase in the psychosexual stage of development, during which sexual activity and the ingestion of food were composite. In her interpretation of Hua mortuary anthropophagy destructive psychic energy was released, separating psychological states from a primordial unity (Sanday, 1986, p. 48).

Freud regarded eros, the instinct that bound people together, and death, the instinct of aggression, as mutually opposed but composite in social ambivalence, which expressed love and hate towards the same object (Sanday, 1986, p. 49). Sagan (1974) suggested that emotional ambivalence motivated acts of cannibalism, as the undeveloped imagination of cannibals was unable to sublimate their oral aggression (though the ethnographic data in the present paper repudiate this assumption) (Sagan, 1974, p. 27). Sanday suggested that emotional ambivalence was apparent in the over-arching ontology that motivated anthropophagy, confirming identity and controlling natural aggression (Sanday, 1986, p. 50). Amongst the Hua, emotional ambivalence was apparent between certain kin categories, the major distinctions being between consanguines and affines and parent and eldest child (Sanday, 1986, p. 67).

Gillison (1993) based her analysis of Gimi mortuary anthropophagy primarily on myths of origin (Leavitt, 1994; Lipset, 1994). She suggested that Gimi life-cycle rituals gave rise to primordial sexual fantasies of incest and intrigue, which threatened a person’s development. This threat was countered through rituals whose themes were apparent in Gimi male and female myths, which focused on women’s role in the origin of social life and, by extension, why women were objects of male exchange (Leavitt, 1994, p. 479). Male myths and fantasies had counterpart female myths and fantasies, male ones based on the male Oedipus complex and female ones on the female Oedipus complex (Gillison, 1993, p. 167n6). Males resolved their Oedipal encounters and homosexual fantasies through female anatomy and character as portrayed in myths, overcoming their ambivalence to the father figure by blaming women (Gillison, 1993, p. 220).

Mortuary anthropophagy was depicted in contradictory male and female myths; the men’s myth depicted woman as a rapacious widow seeking new husbands to devour, whilst the women’s myth depicted woman in an incestuous marriage, which she escaped from by tricking her husband into eating his first-born child (Lipset, 1994, p. 166). Gillison claimed that:

…women’s ritual fury, frenzy, and appetite derive not from simple grief nor a wish to spare the dead a slow decay, as women declare, but rather from the logic of an incestuous drama, a mythic first marriage that drove woman to murder her husband - or his child – out of a complex mix of guilt and revenge. (Gillison, 1993, p. 97)

In the men’s myth of the bamboo flutes, men stole the bamboo flutes, which symbolised fertility, from women, who in return consumed men’s corpses during mortuary rituals to recapture the phallic power originally stolen by men. The destructive antisocial behaviour of women during mortuary anthropophagy was countered by male exchange, forcing the women to release the spirit of the dead. Both sexes struggled to control phallic power, power that was manifested in the vital substances of *auna* and *kore*, to reconstitute the primordial androgenous state, a struggle portrayed in myth and ritual that manifested in sexual antagonism (Sanday, 1986, p. 77). This sexual conflict determined the dichotomy of the Gimi universe (Gillison, 1980, p. 144). In comparison to the traditional mortuary rites of the South Fore (Whitfield et al., 2015), who live adjacent to the Gimi, Gillison’s (1993) post-anthropophagic ethnographic narrative of Gimi mortuary rituals allows an interpretation which highlights female aggression.

Stephen (1998) argued that mortuary cannibalism, analysed from a Kleinian perspective, involved a complex interaction between unconscious guilt and the need for reparation, which created powerful cannibalistic urges. In the case of the Gimi, Stephen argued that cannibalism was based on a powerful unconscious desire to consume the mother.

Gillison (1993), Sanday (1986) and Stephen (1998) gave psychosexual theoretical interpretations that emphasised aggression at the expense of indigenous explanations and removed mortuary anthropophagy from the analytical context of mortuary rites. However, in a paper on the mortuary rites of the Ankave of the eastern highlands of Papua New Guinea, Pierre Lemonnier (2007) brought together Hertz (1960) and Freud (2011) through the work of Lagache (1993) to analyse the presence of cannibal spirits, known as *ombo*, during Ankave mortuary rituals and why the mourners chased off the spirits of the dead. His work showed the absolute parallel between ambivalence and the length of the intermediary period in Ankave mortuary rites, which did not involve actual anthropophagy (Lemonnier, 2006; Lemonnier, 2007, p. 34). Given Conklin’s helpful insights into mortuary anthropophagy we have followed Lemonnier’s lead and interpreted South Fore mortuary rites using Hertz’s theory of secondary burial and Lagache’s understanding of the mourning process to see if aggression, beyond that experienced during other forms of disposal of the body, occurred during mortuary anthropophagy.

We do not use the term ‘cannibal’, which connotes the imaginary Western cannibal found in literature, film and media (Brown, 2013), but instead use the term ‘anthropophagic practices’ to refer to all acts of consumption of the dead or their bodily substances (Whiteley, 2008). The term ‘transumption’ refers specifically to the traditional mortuary rites that involved anthropophagic practices and rituals in the eastern highlands of Papua New Guinea (Whitfield et al., 2015), and is defined as: “the mortuary practice of consumption of the dead and incorporation of the body of the dead person into the bodies of living relatives, thus helping to free the spirit of the dead.” (Alpers, 2007).

## The Theoretical Works of Hertz and Lagache

Robert Hertz’s ‘Contribution à une Étude sur la Représentation Collective de la Mort’, published in English as ‘The Collective Representation of Death’ (Hertz, 1960), was first published in 1907 in *L′ Année Sociologique*. He skilfully combined ethnographic data with theoretical analysis, drawn from the Durkheimians, a group of sociologists associated with Émile Durkheim (2006), to write about primary and secondary mortuary rites and associated behaviours and beliefs. Following death society’s continuity was accomplished through the transition of the deceased, achieved through secondary burial. The primary rites separated the flesh and the deceased’s identity; the secondary stage gave the deceased a new identity as an ancestor, thus recreating an ideal society (Davies, 2000). The intermediary period started from the moment of death and lasted till the final ceremony, a period marked by the separation of the flesh from the bones, and a transition in the condition of the souls of the deceased and the mourners. During the final ceremony a great feast was held to celebrate the life of the deceased, the physical remains of the deceased were placed in their final sepulture, the souls of the deceased were reunited in the land of the dead as an ancestor, and the living were freed from the obligations of mourning (Hertz, 1960, p. 54).

Hertz believed that death was caused by the intervention of negative forces, destroying the social being grafted upon the physical individual and threatening the permanence of society and its faith in itself (Hertz, 1960, p. 77). For society to overcome death the deceased was reborn as an ancestor, whilst certain aspects of the deceased were kept by society (Hertz, 1960, p. 78). Furthermore, a psychological theme ran through Hertz' work, though he treated it as a sociological discourse, which related the length of time of the intermediary period to the time it took for the body to decompose and to the period of mourning.

Lagache (1993) recognised the “work of mourning” in both pathological forms of mourning and in the rituals of indigenous societies allowing the function of mourning to be analysed from both a psychological and social basis (Lagache, 1993, p. 15). The aim of mourning was to achieve the separation of the dead and the living, achieved through the recognition on the human level of the biological fact of death described by Lagache as “killing the dead” (Lagache, 1993, p. 17). Following the death of a loved one a person experienced grief, manifested as a loss of interest in their surroundings and in people, whilst their attention was focused on the deceased. At first the mourner experienced both the presence and absence of the deceased, with absence gradually overcoming presence until the deceased was finally bade farewell. As grief faded the mourner re-engaged with society.

In the morbid forms of mourning Lagache was able to penetrate the structures of mourning more deeply, and recognised exaggerated phenomena that occurred in normal mourning: the inability of the mourner to recognise death, having dreams and visual and auditory illusions maintaining the presence of the deceased, identification of the mourner with the state of the deceased, feelings of guilt and responsibility for the death of the deceased, a deceased who was now idealised, and, finally, feelings of ambivalence towards the deceased, which manifested from feelings of love and hate towards the person when they were alive. The recognition of the phenomena enabled him to identify three of the mechanisms of mourning: the unacceptability of death, ambivalence towards the deceased and identification with the deceased (Lagache, 1993, p. 18). Lagache was then able to identify the three mechanisms of mourning in the work of Hertz (1960).

The unacceptability of death identified by Lagache had a psychological and social aspect. The psychological aspect was apparent in the mourning process of the close family members, and the social aspect in the mortuary rituals. Both processes gradually transformed the unacceptability of death to the acceptance of death.

Ambivalence, the second mechanism, consisted of the loving emotions expressed by the mourners as conscious grief, and hostile emotions, which arose from the unconscious satisfaction of death, expressed as aggression.

The third mechanism was identification with the deceased, which was apparent in the way the deceased was treated as though he was living, and the relatives as though they were dead. The deceased continued to maintain rights over his widow, whilst the living, contaminated by death, were isolated from the community. Identification was also achieved by wearing relics of the deceased and through the practice of endocannibalism, during which the deceased’s powers and body were absorbed, and the body placed in an appropriate sepulture (Lagache, 1993, pp. 26-27).

Lagache recognised three sources of aggression in the work of mourning: aggression based in all human relationships -- this aggressive component freed at death was projected onto the deceased, manifesting as an evil spirit or demon; resentment towards the deceased for abandoning a loved one -- this was the mourners’ resentment for the deceased dying, which manifested as blame of the deceased, family members or sorcerers; and aggression in the destructive work of mourning, which manifested in the isolation of the deceased and their relatives and the destruction of the body, so the deceased could reach the afterlife (Lagache, 1993, p. 25). Following biological death, the projected aggression of mourning was gradually diffused during the intermediate period, allowing the deceased to transform into a benevolent ancestor during the secondary burial, which finally achieved death on a social level (Lagache, 1993, p. 26).

This paper focuses on the three mechanisms of the mourning process and their associated forms of aggression, as described by Lagache (1993), to show that transumption was a form of secondary burial that alleviated the primary concerns of the Fore in relation to the disposal of the corpse of a loved one, and did not entail aggression beyond that of the work of mourning experienced in every form of disposal of the body.

## Ethnographic Descriptions of Traditional Mortuary Practices of the Fore People

### Data Collection

The following ethnographic descriptions are based on transcriptions and field notes collected between 1996 and 2010. The transcriptions were based on interviews collected from 68 men and 20 women, belonging to 20 different villages and 22 different clans from the South Fore. The elderly interviewees witnessed the traditional mortuary rites during childhood when they attended funerals with their families. In the case of transumption the children attended the funerals with their mothers. Males participated in the practice of transumption until they underwent the first stage of initiation, aged between 6 and 8 years, after which they no longer participated in the practice. This meant that males and females had nearly equal childhood witness to the practice of transumption (Whitfield, 2011). The gender imbalance of the interviewees was the result of the kuru epidemic, a fatal neurological disease that affected the Fore and their neighbours, which declined after the cessation of transumption at the end of the 1950s (Alpers, 2007). In this paper we use terms in the *pamusakamana* dialect to refer to concepts that apply equally to the *atikamana* and *pamusakamana* dialects of the South Fore (Scott, 1963). Claims by some anthropologists that transumption never occurred have been proven to be unfounded (Whitfield et al., 2015).

### The 5 Souls of the South Fore

A detailed ethnographic description of the South Fore cosmology, beliefs regarding the five souls and traditional mortuary rites, including transumption, has already been published elsewhere (Whitfield et al., 2015), so we include only a brief ethnographic account of these beliefs and practices in this paper.

At conception a child was formed from the father’s sperm and the mother’s blood. The patrilineal substance formed the bones and the *auma* and *ama* of a person; the *auma* was described as similar to the Western concept of the ‘I’, but consisted of only the good qualities of a person; the *ama* was a simulacrum of the *auma*, with additional magical powers to assist relatives after death. The matrilineal substance formed the flesh and blood, which transformed into the *kwela* at death, endangering the family members if the mortuary rites were not performed correctly. By the end of the mortuary rites the *kwela* consisted of only patrilineal blood and no longer presented a danger to the family members (Whitfield, 2011, pp. 190-191).

The South Fore person consisted of 5 souls; after death the souls were dispersed, but the *auma*, *ama* and *kwela* were eventually reunited in the land of the dead, called *kwelanamandi*, where the deceased was reborn as an ancestor. The *aona* and *yesegi* were inherited by one of the deceased’s children. The *aona* was a person’s abilities and male and female initiates received *aona* during rites of passage. The *aona* took the form of a familiar spirit and in some cases it had a counterpart called *yesegi*, the aggression, lack of fear and supernatural power of the *aona*. After death the *ama* also bestowed *aona* and *yesegi* on mourners who expressed their love for the deceased (Whitfield, 2011, pp. 177–190).

### Death

Death marked the start of Hertz’s intermediary period, which continued until the final ceremony called *agona* in the South Fore. During this time the close mourners of the deceased were isolated from society, the corpse decomposed, removing the flesh from the bones, and the deceased’s *kwela* was active. This period was marked by a gradual change from the time of death to the final ceremony, as restrictions on the mourners were gradually lifted, the threat of harm from the *kwela* gradually faded and the danger posed by the corpse declined.

After death the deceased’s affines arrived in the village. Leading the way came the *anagra*, the married female affines, followed by their menfolk armed with bows, arrows and axes, and lastly the young and old. The men raided the deceased family members’ gardens and killed their pigs, stacking the food outside the men’s house, where the family members added additional items as part of the compensation payment for the death. The violence displayed was an expression of the affines’ love for someone they shared their blood with, and punishment of the family for allowing a person they loved to die. The damage committed was equated to the social standing of the deceased. If a child died the affines would beat the family members with thorns and nettles for not taking care of the child properly and this allowed the affines to vent their grief, frustration and anger over an untimely death. Sometimes these actions led to fighting between the mourners; however, this was expected behaviour, and soon they would lay aside their differences and mourn together. Further outbursts of violence by the affines occurred until the final mortuary ceremony was completed, but these outbursts were ways of showing affection for the deceased, and the deceased’s family would have been offended if they had not occurred. The love expressed by the affines reduced the grief of the family members, when they realised they were not mourning alone, and this helped them to forget the deceased, which was necessary if the *ama* and *kwela* were to eventually depart to *kwelanamandi* (Whitfield, 2011, pp. 198–203).

The body was dressed in the deceased’s finest attire, the skin oiled with pig fat, and then placed on a bier of red sugarcane with the body sat upwards at forty-five degrees and the head supported horizontally with wood. This allowed the mourners to remember the deceased as he was when living and prepared the *auma* to meet the ancestors in *kwelanamandi* (Whitfield, 2011, pp. 203–205).

A man’s bier was placed on the border of the men’s and women’s areas of the village, whilst a woman’s was placed in the *kami*, the women’s area of a village. The mourners gathered around the body and cried, slept and sang depending on how they felt. They sang songs about the deceased, the land, and the road to *kwelanamandi*; some were soothing whilst others motivated revenge. The *ama* and *kwela* were present during the singing and the mourners sang to say farewell to the deceased and to let him know of their love and sorrow (Whitfield, 2011, pp. 208–211).

Two words were commonly used to describe grief: *kayala,* which was the physical pain experienced from a burn, and *kaisukaisu,* which referred to mental pain. The intensity of grief depended on the individual relationship between the mourner and the deceased. A father was called *tumu waga,* the name of a house’s centre post. If the post collapsed the house collapsed, symbolising the effect on a family if a father died. If a roof collapsed the posts remained standing; the roof symbolised the loss of a mother and the posts the remaining members of the family without the mother’s protection. If a daughter died there was no food and water and the family went hungry, and if a son died there was no firewood and the family were cold (Whitfield, 2011, pp. 230-231).

During the night an age-mate of the deceased would tie some of the deceased’s hair to an arrow, light it, and fire it towards the clan’s *amani*, the sacred ground (with the same name) of the clan’s *amani* (guardian ancestral spirit), alerting the *amani* to the death of a clansman and requesting his help to avenge the deceased. The *bakana* ritual was then performed to find the sorcerers responsible for the death; among the South Fore nearly all deaths were attributed to sorcery. A male member of the family consumed hallucinogenic plants and hyperventilated on tobacco resulting in his possession by the deceased’s *ama,* and guided by the *kwela* he would attempt to identify the responsible sorcerers. During the same evening a senior male relative would hang up some of the deceased’s clothes in the *amani*, and request the sacred ancestor’s assistance to guide the *auma* to *kwelanamandi.*

As a man lay dying his spirit familiar, received from the *bagina* (the land and creator), came and grieved over him before dying itself, severing the link between the *bagina* and the dying man. Then, just before death, the dying man would tell his relatives that the ancestors had arrived to escort him to *kwelanamandi*. After death the *auma* departed from the body, crossed the land and said goodbye to the *bagina.* When the *auma* arrived at the *amani* it explained to the *amani* why he had died and after being fed was shown the road to *kwelanamandi*. The *auma* was accompanied by the ancestors on the dangerous journey to *kwelanamandi* during which they overcame wild ghosts called *kavugi* which inhabited the road. After three days the *auma* crossed the red river at the entrance to *kwelanamandi* and was welcomed by the ancestors. This event coincided with the appearance of the first signs of decomposition of the corpse, heralding the *auma*’s safe arrival in *kwelanamandi*, where the *auma* remained on its allocated land awaiting the arrival of the *kwela* and *ama*.

Once the first signs of decomposition occurred the body was carried to a sepulture designated by the deceased, which was normally marked by a bamboo or wild sugarcane grove. The mortuary rites so far were very similar for males and females, and were of a more limited nature for children; however, they were the same for all forms of caring for the body.

### The Last Acts of Caring for the Body

#### Transumption

A man’s body belonged to his mother’s brothers’ clan and a senior uncle was appointed to oversee its handling and proper care. In turn he appointed his wife to oversee the transumption of the body, whilst he witnessed events to ensure that the *anagra*, the adult female affines, performed their duties with love and respect. The deceased’s oldest son’s wife would make a request on behalf of the *anatu*, the adult women of the deceased’s clan, for the head and right arm of the deceased. The arm symbolised the deceased’s support and protection for his family and the head symbolised his individual humanity.

A woman’s body was claimed by her oldest brother, who transferred the rights to his cousins’ wives, one of whom oversaw the distribution of the body. Again, the *anatu* would request an arm and the head of the deceased. The rights to an unmarried woman’s or girl’s body belonged to her eldest brother, who transferred them to his cousins’ wives, one of whom would oversee the distribution of the body.

The body was taken to the chosen sepulture, normally a bamboo or sugarcane garden or casuarina grove which had been a favourite place of the deceased when he was alive. The body was placed on a bed of wild green vegetables, on a tapa cloth on top of banana leaves. The close family members did not normally stay to witness this event as it was too traumatic for them. Occasionally a man from the family appointed to witness the event would shoot one of the *anagra* with an arrow as she dismembered the body. He would use a non-barbed arrow and shoot the woman in the thigh, the traditional site used to punish women by men. This was an expression of a man’s love for the deceased and his family would later compensate the woman’s family for the injury.

The senior *anagra* made the first cut, and as the first drop of blood fell from the body, the *bagina* welcomed and comforted the *ama* and *kwela*. The flesh was placed in piles with corresponding bones on edible ferns. The women then proceeded to cut the flesh and intestines into smaller pieces and cooked them in bamboo tubes on a fire made at the site of dismemberment.

Once cooked, half of the flesh was placed onto a communal leaf plate to be distributed amongst the *ename*, who were unrelated women from surrounding villages who came to mourn the deceased. The rest of the flesh was consumed by the *anatu* and *anagra* and their female relatives and children, and as evening approached they took the remaining flesh to the mourning house and consumed it during the night.

The following morning, in a rite called *ikwaya ana*, the *anatu* and *anagra* took the remaining flesh and bones back to the sepulture. The dry bones were wrapped in wild green vegetables and carefully crushed, then placed in bamboo tubes, cooked and eaten. As evening approached, the hard bones such as the skull and shoulder blades were placed in the fireplace and covered with earth; this became the sepulture and the temporary residence of the *ama*.

The women returned to the mourning house and remained there until all the bones were consumed. When transumption was completed the first purification rite called *pepatakina* was performed. Vegetables were cooked in an earth oven and the steaming leaves from the oven placed outside the mourning house. As the women walked out onto the leaves the steam partially purified their bodies allowing them to prepare for the next purification rite called *aindu*. The women hunted garden rats, whilst the men hunted wild animals. The animal carcases were taken to the mourning house, gutted and placed on the fire and the strong smell of burning fur and fat purified the village. The women rubbed the burnt carcases on their bodies and anywhere else they might have touched during transumption, thus removing any pollution and ensuring that all the body was consumed. The carcases were then eaten by the women.

*Kavunda* took place over one to two months and was a time of intense grieving. It was the time between the completion of transumption and the time when grass started to grow on the deceased’s sepulture, marking the start of preparations for *aluana*. *Aluana* was the last purification feast for the participants of transumption, and also thanked them for eating the body. The women hunted garden rats and collected grubs whilst the men hunted wild animals. The food was distributed to all the guests but the focus was on the women who had participated in transumption. *Aluana* marked the departure of the *kwela* from the bodies of the *anagra* to the sepulture, where it joined the *ama*. When enough pigs were available a payment called *igoghana* was made to the families who consumed the body.

Preparations for Hertz’s final ceremony, called *agona* in the South Fore, took two to three years and might require up to twenty-five pigs depending on the social status of the deceased. The main payment went to the mother’s brothers’ clan and the wife’s clan in a man’s case, and to a woman’s patrilineal clan in a woman’s case. During *agona* all the deceased’s social relationships were transferred from the deceased parent to their children reproducing the social aspect of the person and indirectly the clan. The *ama* and *kwela* departed saying farewell to the *bagina* and to the *amani,* who fed them and showed them the road to *kwelanamandi*. When they arrived in *kwelanamandi* these two souls were reunited with the *auma* and the deceased was reborn as an ancestor. Finally, the mourners were reintegrated into society.

Human bodies were not meant to be eaten because of the pollution they contained, but the women ate them anyway out of love and affection for the deceased, and because they did not want the body to be eaten by worms if it was buried, or maggots if it was placed on a platform or in a basket. The grief of the mourners peaked during the dismemberment of the body and subsided after they consumed the body. The mourners felt that the deceased was part of them after transumption and this alleviated their grief. Furthermore, the *kwela* remained inside the *anagra* until *aluana*, protecting the family from its presence, whilst the *ama* remained with the family. Even though the person had died their souls still remained with the living, and had not yet departed; they only departed when the mourners’ grief had subsided and they had accepted the death. Furthermore the women taunted the men as they ate the body asking them why they were eating the body of a loved one and the enemy were not. The men were angered by the situation and promised the women that they would avenge the death, and this also alleviated their grief. The taunting occurred during all forms of disposal of the body and was not behaviour specific to transumption.

#### The Widow and Widower

The widow was dressed in attire to mark her mourning. She wore the bark cape her husband’s body had been placed on and carried old bilums (net bags), bush rope was tied to her limbs, and her skin was covered in red clay. She would abstain from her husband’s favourite foods and his favourite place to show her love for him. If her grief was intense she burnt his possessions and consumed the ashes with wild vegetables during *kavunda*, and this eased her grief. The widow grieved in the presence of the *ama* and *kwela* and the presence of the *kwela* made her polluted and a threat to the community, so she was isolated during the day by her latrine and during the night stayed in her house. After *aluana* she was able to move around the village but remained in close contact with the *kwela*. After 2-3 years she could remarry if she chose to and in a rite called *kilanu kasa* she was freed from her husband’s *ama* and *kwela*.

A widower remained in the men’s house during the mourning period and after 2-3 years would remarry if he chose to. His wife’s *ama* remained with him during this time and her *kwela* did not pollute him. When he started to forget his wife as his grief faded it was said that his wife’s *ama* had departed to *kwelanamandi.*

#### Burial

Adults sometimes chose to be buried if they loved their land and wanted to be eaten by it. Grave pits could be dug near human settlements as most of the pollution from the decomposing body was contained in the pit. The grave was round and narrow, its sides reinforced with woven sticks and cordyline leaves and the bottom covered with cordyline leaves and a piece of tapa cloth. The body was placed in a sitting position with the back leaning against a piece of wood and a piece of wood under the jaw kept the head upright. The body was placed facing the deceased’s favourite creek or other place important to him. Finally the grave was filled with cordyline leaves, covered with wood and soil, and a fence was built around the sepulture to protect the body from pigs and dogs. Food and water were placed at the graveside for the next three days for the *ama* and *kwela*, but after that the *kwela* became more powerful as the body continued to decompose and the grave was avoided until it was time to perform *isakava galo*.

*Isakava galo* was performed after the collapse of the burial pit 5-6 years later, and released the *ama* and *kwela* from the grave. Burial allowed for the flesh to be eaten by the *bagina* leaving only the bones. During the final ceremony a secondary sepulture was prepared, consisting of a wicker basket 1.5 m high, the top of which was covered in orchid leaves to hold the bones. A maternal uncle of the deceased opened the grave and poured in a libation of pig’s blood to please the *ama* and *kwela,* so they would be happy for the bones to be removed. The bones were removed, placed on a tapa cloth, rubbed with pig’s blood or grease, and placed on the new sepulture. The *ama* and *kwela* were told that they had been released from darkness into light and they remained at the new sepulture seeing their land until their final departure. Eventually the secondary sepulture collapsed and this symbolised the departure of the *kwela* and *ama* to *kwelanamandi.* In the case of burial *agona* was normally performed 2-3 years after death, as the affines would be angry if they had to wait 5-6 years for the *agona* payment (Whitfield, 2011, pp. 724–730).

#### Basket

A basket was constructed and placed in a bamboo grove 2 m above ground with a grass roof covering the structure. The body wrapped in tapa cloth was placed inside and covered with leaves. A dish-shaped hole was dug under the basket to ensure that the fluids of decomposition entered the ground at a single point and were consumed by the *bagina*. Relatives of the deceased would light a fire near the sepulture to keep the *ama* and *kwela* warm and place food and water by the bamboo grove to feed them. After 2-3 days the decomposition of the body started to transform the flesh and blood into the dangerous *kwela*, and the family stayed away until decomposition was complete. Basket sepultures were always built away from the village because of the fear of the *kwela*, and because of the large number of flies attracted to the corpse. When the family was ready they performed *agona*, which in the case of basket sepultures marked the final ceremony. Finally the bones fell into the bamboo grove and this symbolised the departure of the *kwela* and *ama* to *kwelanamandi* (Whitfield, 2011, pp. 732–734).

#### Platform

Disposal of the corpse on a platform was very similar to that of a basket except for the actual sepulture. The platform was constructed from wood covered with tapa cloth at a height of 3 m above the ground; it had walls, which were constructed of saplings, and a roof of grass. The corpse, wrapped in tapa cloth, was placed in the miniature house. If the bones were retrievable *isakava galo* was performed at the same time as *agona*; otherwise *agona* was performed on its own and marked the departure of the *kwela* and *ama*.

#### Removal of Bodies From Sepultures

Sometimes the *anagra* and *anatu* decided to remove the body from a sepulture and to eat it out of love and respect for the deceased. They would ask the deceased’s brothers, who then assisted the women in removing it. The body was distributed as for transumption. Occasionally the *anagra* decided to remove the body secretly, but the male family members always knew in advance and removed their men who guarded the sepulture before their arrival. The *anagra* approached the sepulture during the night and told the *ama* and *kwela* that their family did not care for them and they had come to consume the body. The older women dismembered the body and carried it back to the mourning house, where they consumed the flesh and on the subsequent night the bones. The jaw and collar bones were placed back on or in the sepulture. The *ama* and *kwela* knew that the women had eaten something which was not fit to be consumed and felt sorry for the women. When the *anagra* were ready they told the male family members, so *aluana* and subsequent rituals could be performed. The male family members were not cross with the women as they knew the deceased’s *kwela* was inside them. Nobody was angry about the theft of the body, as it was removed and consumed out of love and respect, and with the collusion of all parties concerned.

## Conclusions

The three mechanisms of mourning, identified by Lagache (1993), were apparent in all forms of traditional corpse handling and care employed by the South Fore. The unacceptability of death to the mourners and society was gradually overcome; on a psychological level absence gradually overcame presence till grief faded and the mourners re-engaged with society; on a social level death was overcome through the rebirth of the deceased as an ancestor and the inheritance of the deceased’s identity, confirming the immortality of the person and society.

The ambivalence of mourning was experienced through conscious grief and unconscious aggression; the latter was apparent in the inverted hostility of the living to the deceased matched by the aggression of the deceased to the living in the form of the *kwela*. The concept of the *ama* was a manifestation of the mourner’s disinvestment in the external world and investment in the internal world in an attempt to keep the deceased alive. The mourner continued to exchange acts of love with the *ama* throughout the mortuary rites, and afterwards with their ancestor through relics, which acted as a substitute for the deceased.

Identification was further achieved through the isolation of the family members, especially a widow, after the death of a close consanguineal member. The deceased’s *ama* and *kwela* continued to interact with the family during mourning, and the deceased was treated as though he was still alive in certain ways. The *aona* and *yesegi* of the deceased were inherited by the deceased’s children linking the children through their parent to their ancestors and clan *amani*, and ultimately to the *bagina.* After transumption female participants stated that the deceased had become part of them, and some participants received *aona* from the deceased’s *ama* for having eaten part of the deceased out of love and respect. The three sources of aggression in the work of mourning were equally apparent in all forms of corpse disposal. The aggression in human relationships was projected from the mourners onto the deceased in the form of the *kwela*, the dangerous soul formed from the matrilineal flesh and blood of the deceased. Resentment towards the deceased dying was apparent in South Fore sorcery beliefs, which blamed nearly all deaths on enemy sorcerers (Lindenbaum, 2013). The destructive work of mourning was necessary for the deceased to be reborn as an ancestor; this was apparent in the isolation of the deceased’s body, in the behaviour of the relatives, particularly the widow, and in the destruction of the body.

All forms of corpse destruction practised by the South Fore resulted in the removal of the flesh from the bones and opened the door for the *kwela* and *ama* to depart to *kwelanamandi*. Bloch and Parry made the general observation that societies which distinguish between kin and affines are also likely to distinguish between male bone and female flesh and to separate them at death. Furthermore, they suggested that these distinctions concluded in the final ceremony with a triumph over the need for affines and the sexual reproductive capacity they provided (Bloch & Parry, 1982, p. 21). Their observation was pertinent for the South Fore, where the separation of the flesh resulted in the symbolic triumph over the need for affines and the ambiguous relationship they presented (Glass, 2011; Lindenbaum, 2013).

The South Fore emphatically stated that they ate the bodies of their loved ones out of love and respect, and we found no ethnographic evidence to support any suggestion of explicit or implicit aggression during transumption, beyond that experienced during the work of mourning for all forms of disposal of the body. Regardless of social status, the decomposition of the body of a loved one was a prime motivating force behind transumption for the South Fore, who did not want their loved ones’ bodies to be eaten by maggots or worms. They preferred to place them in the most honourable sepultures available – their own bodies.

These specific findings for the traditional mortuary rites and mourning rituals of the South Fore people affirm the relevance of the concepts of Hertz and Lagache to these rites and rituals and, more generally, to the universal human experiences of death and mourning.
